# Supplementary material for: Characteristics of Microstructural Changes Associated with Glioma Related Epilepsy: A Diffusion Tensor Imaging (DTI) Study
Source: Brain Sci. 2022 Aug 31;12(9):1169. doi: 10.3390/brainsci12091169 (PMC9496781; doi:10.3390/brainsci12091169)
Supplement: Supplementary file 1 [file brainsci-12-01169-s001.zip › brainsci-1826279-supplementary.pdf]

Supplementary material

Supplementary Table S1.-Demographic and clinical characteristics

Patient No.	Age (years)	gender	Histopathological type	Tumor volume(mm^3)
Sub010	50	F	Astrocytoma, WHO grade II	27.34
Sub018	41	M	Glioblastoma , WHO grade IV	127.40
Sub062	33	F	Astrocytoma, WHO grade II	34.11
Sub071	27	F	mixed glial neuronal tumor，WHO grade I	3.97
Sub072	48	M	Anaplastic astrocytoma , WHO grade III-IV	54.88
Sub081	36	F	Oligoastrocytoma, WHO gradeII	19.64
Sub087	54	M	Oligodendroglioma , WHO grade II	41.24
Sub100	44	M	Oligoastrocytoma, WHO gradeII	7.76
Sub102	51	M	Oligodendroglioma , WHO grade II	40.78
Sub124	51	M	Astrocytoma, WHO grade II	20.42
Sub125	19	M	Ganglioglioma，WHO grade I	2.61
Sub176	65	M	Oligoastrocytoma, WHO gradeII	22.02
Sub004	45	M	Glioblastoma ，WHO grade IV	115.91
Sub017	51	M	Anaplastic oligodendrocytoma, WHO grade III	39.70
Sub024	36	F	Oligodendroglioma , WHO grade II	24.10
Sub030	39	M	Oligoastrocytoma, WHO gradeII	9.32
Sub061	45	F	Glioblastoma , WHO grade IV	49.31
Sub063	56	M	Anaplastic astrocytoma , WHO grade III	26.47
Sub082	46	F	Oligoastrocytoma, WHO gradeII	58.86
Sub127	45	F	Glioblastoma , WHO grade IV	3.72
Sub138	50	M	Oligoastrocytoma, WHO gradeII	37.27
Sub152	49	M	Anaplastic astrocytoma , WHO grade III	22.28

Sub162	35	F	Oligodendroglioma ， WHO grade II	86.40
Sub174	51	M	Astrocytoma， WHO grade II	65.24
Sub175	45	F	Anaplastic oligodendrocytoma， WHO grade III	30.69

Supplementary Table S2.-Two studies related to GRE[1, 2]

First author	Publish year	Tumor type	Patients
Yinyan Wang	2015	low-grade glioma	410
Mao Yunhe	2020	histologically confirmed gliomas	119

1. Yunhe, M., et al., *Mapping seizure foci and tumor genetic factors in glioma associated seizure patients.* J Neurosurg Sci, 2020. **64**(5): p. 456-463.
2. Wang, Y., et al., *Localizing seizure-susceptible brain regions associated with low-grade gliomas using voxel-based lesion-symptom mapping.* Neuro Oncol, 2015. **17**(2): p. 282-8.
